# The *AIRE* -230Y Polymorphism Affects AIRE Transcriptional Activity: Potential Influence on AIRE Function in the Thymus

**DOI:** 10.1371/journal.pone.0127476

**Published:** 2015-05-15

**Authors:** Thomas R. J. Lovewell, Andrew J. McDonagh, Andrew G. Messenger, Mimoun Azzouz, Rachid Tazi-Ahnini

**Affiliations:** 1 School of Medicine and Biomedical Sciences, University of Sheffield, Sheffield, United Kingdom; 2 Dermatology Department, Royal Hallamshire Hospital, Sheffield, United Kingdom; 3 Department of Neuroscience, School of Medicine and Biomedical Sciences, University of Sheffield, Sheffield, United Kingdom; Universidade de Sao Paulo, BRAZIL

## Abstract

**Background:**

The autoimmune regulator (AIRE) is expressed in the thymus, particularly in thymic medullary epithelial cells (mTECs), and is required for the ectopic expression of a diverse range of peripheral tissue antigens by mTECs, facilitating their ability to perform negative selection of auto-reactive immature T-cells. The expression profile of peripheral tissue antigens is affected not only by AIRE deficiency but also with variation of AIRE activity in the thymus.

**Method and Results:**

Therefore we screened 591bp upstream of the *AIRE* transcription start site including *AIRE* minimal promoter for single nucleotide polymorphism (SNPs) and identified two SNPs -655R (rs117557896) and -230Y (rs751032) respectively. To study the effect of these variations on *AIRE* promoter activity we generated a Flp-In host cell line which was stably transfected with a single copy of the reporter vector. Relative promoter activity was estimated by comparing the luciferase specific activity for lysates of the different reporter AIRE promoter-reporter gene constructs including *AIRE*-655G *AIRE*-230C, *AIRE*-655G *AIRE*-230T and *AIRE*-655A *AIRE*-230C. The analysis showed that the commonest haplotype *AIRE*-655G *AIRE*-230C has the highest luciferase specific activity (p<0.001). Whereas *AIRE*-655G *AIRE*-230T has a luciferase specific activity value that approaches null. Both *AIRE* promoter polymorphic sites have one allele that forms a CpG methylation site which we determined can be methylated in methylation assays using the M.SssI CpG methyltransferase.

**Conclusion:**

*AIRE-230Y* is in a conserved region of the promoter and is adjacent to a predicted WT1 transcription factor binding site, suggesting that *AIRE-230Y* affects AIRE expression by influencing the binding of biochemical factors to this region. Our findings show that *AIRE*-655*GAIRE*-230T haplotype could dramatically alter AIRE transcription and so have an effect on the process of negative selection and affect susceptibility to autoimmune conditions.

## Introduction

Self tolerance processes in the thymus play a key role in thymocyte maturation and in preventing the development of auto-reactive T-cells, and consequential autoimmune reactions. Negative selection of immature thymocytes with auto-reactive potential is an important part of this process and in order to be effective, requires the body’s repertoire of self-antigens to be presented to these immature cells. This task is carried out in the medullar region of the thymus, predominantly by the presentation of peripheral tissue antigens (PTAs) by medullary thymic epithelial cells (mTECs) and dendritic cells (DCs) to immature thymocytes [1]. The Autoimmune Regulator (*AIRE*) gene plays a key role in this process of negative selection by regulating the promiscuous expression of PTAs in mTECs [1, 2].

Autoimmune Polyglandular syndrome 1 (APS-1) is a monogenic disorder caused by loss-of-function mutations in the *AIRE* gene [3, 4], characterised the diagnostic triad of adrenal insufficiency, hypoparathyroidism and chronic mucocutaneous candidiasis [5]. APS-1 patients have an elevated incidence of secondary autoimmune conditions, such as type 1 diabetes, alopecia areata and pernicious anaemia [6], suggesting a potential link between *AIRE* and susceptibility to these secondary autoimmune conditions.

The level of Aire expression has been shown to have functional effects in several studies using murine models [7, 8]. An early report comparing wild type mice with homozygous (-/-) and heterozygous (+/-) *Aire* knockout mice observed that both T-cell deletion in the thymus reduced and incidence of diabetes increased as the amount of Aire decreased [7]. Other studies have discovered that the expression of Aire-regulated promiscuously expressed genes are also affected as the amount of Aire expression decreases [8], with a recent investigation using siRNA demonstrating that Aire knockdown affects the expression of peripheral tissue antigens both *in vitro* and *in vivo* [9]. Here, we sort to determine whether polymorphisms in the promoter region immediately upstream of the *AIRE* gene, affected its transcriptional activity, and thus could have a functional downstream effect.

## Materials and Methods

### Transgenomic WAVE dHPLC screening of the *AIRE* promoter

The first 591bp of the *AIRE* promoter was split into two regions for screening and polymerase chain reaction (PCR) employed to amplify each region. The first region (Region 1; 472bp) was amplified using primers EIF3 (CTCAGAGAAGGAAAGGACTTGCC) and PF REV (TCGCTCTCGGGAAGTCTC) and the second (Region 2: 478bp) using primers HP FOR (GTCTCGGCTTTGCCCCATTCGA) and HP REV (TGCAGCCTCAGAAGCCGGCGTA). For each region, the PCR product from an unknown sample was mixed 50:50 with the PCR product from a control sample of known sequence and PCR products heteroduplexed by heating to 95°C for 5 minutes then reducing the temperature by 0.1°C every 4 seconds until 25°C was reached. Heteroduplexed samples were analysed using a transgenomic WAVE nucleic acid fragment analysis system. Region 1 samples were analysed at temperatures of 62°C, 64°C and 65.4°C and Region 2 samples at 63.3°C, 64.8°C, 65.9°C and 68.6°C.

### PCR RFLP screening of the *AIRE* promoter

Genomic DNA samples from healthy humans were extracted by standard methods from blood samples obtained from the Trent Blood Transfusion Service (Sheffield). All individuals gave informed consent in accordance with approval by the Trent Multi-Centre Research Ethics Committee, Derby.

Our samples had previously been genotyped for the *AIRE*-230Y SNP (single nucleotide polymorphism) [10]. The -655R SNP was genotyped using the primers EIF3 and PF REV (see section 2.1) for PCR amplification, and the subsequent product digested using the restriction enzyme RsaI (New England Biolabs, Hitchin, UK). Alleles were differentiated using agarose gel electrophoresis, with the A allele lacking the restriction site producing a single band of 472bp and the restriction site with the G allele yielding two bands of sizes 384bp and 88bp.

### Generation of reporter gene vectors

The variant *AIRE* promoter haplotypes under study were PCR amplified using primers EIF3 and PIR (ACGCCCCTGGCTCCTTGTG), ligated into a pCR2.1 TA vector (Invitrogen Ltd, Paisley, UK) and then transformed into TOP10 competent cells (Invitrogen). Each promoter variant was subsequently sub-cloned upstream of the firefly luciferase reporter gene in the pGL3-basic vector (Promega, Southampton, UK) to generate pGL3-P(haplotype) *AIRE* reporter constructs for transient reporter gene assays. The *AIRE* promoter and firefly luciferase construct (for all different *AIRE* promoter haplotypes) were then sub-cloned into the pcDNA5/FRT expression vector (Invitrogen), with the *AIRE* promoter replacing the CMV promoter. These pcDNA5/FRT/P(haplotype)*AIRE*-firefly luciferase reporter constructs were used for the stable reporter gene assays. Direct sequencing was used to confirm the identity of the promoters after each cloning step.

### Cell culture

TEC 1A3 cells (a gift from Dr. Garchon, Inserm U580, Paris) were maintained in RPMI 1640 medium (Lonza) supplemented with 10% foetal calf serum (Lonza) and 1% penicillin/streptomycin. Previous research studying the AIRE promoter has been carried out using this cell line [11].

### Generation of TEC 1A3 Flp-In host cell line and stably transfected reporter gene cell lines

A TEC 1A3 cell line containing a single FRT (Flp recombinase target site) was generated using the pFRT/*LacZeo* vector following the Flp-In system manual (012402 version C, Invitrogen). In brief, this process involved the transfection of TEC 1A3 cells with pFRT/*LacZeo* vector using FuGENE6 reagent (Roche). Cells were grown in media supplemented with 250μg/ml Zeocin to select for those containing the pFRT/*LacZeo* vector integrated into the genome, with cloning rings used to isolate foci to collect individual cell lines. Southern blotting was used to screen the different cell lines to identify those containing only a single integrant of the pFRT/*LacZeo* vector. Relative expression efficiency from the FRT region for each TEC 1A3/pFRT/*LacZeo* cell line generated was estimated by comparing β-galactosidase activity using the β-gal assay kit (Invitrogen). The cell line with the highest β-galactosidase activity was chosen to be the TEC 1A3 host cell line for subsequent reactions.

These TEC 1A3 Flp-In host cells were co-transfected with pOG44 with a pcDNA5/FRT/P(haplotype)*AIRE*-firefly luciferase reporter constructs (20:1 ratio) using FuGENE6 reagent (Roche) per transfection. Cells were maintained in media supplemented with hygromycin B at 150μg/ml to select for those containing integrated pcDNA5/FRT/P(haplotype)*AIRE*-firefly luciferase reporter constructs.

### Single integrated vector reporter gene assays

24 hours after seeding into 6-well plates, cells from each promoter haplotype variant of the reporter gene cell lines were washed with PBS before lysis with Glo lysis buffer (Promega). Lysates were assayed in triplicate for luciferase expression using the Bright-Glo assay kit (Promega), using a known concentration of recombinant luciferase (Promega) as a reference sample to normalise luminescence data. Protein concentration (μg/ml) of lysates was quantified in triplicate using the BCA protein assay kit (Perbio). Promoter efficiency was quantified by comparing specific activity (normalised luminescence/protein concentration of lysates) for the different promoter haplotypes.

### Statistical analysis

Difference in relative expression efficiency across the different promoter haplotypes were assessed by one-way analysis of variance (ANOVA) and used Tukey-Kramer multiple comparison test to assess the differences between the individual haplotypes. Analyses were performed using GraphPad PRISM version 5.

### Methylation and bisulphite sequencing of the *AIRE* promoter

The pcDNA5/FRT/P(GC)AIRE-firefly luciferase construct was first methylated using the M.SssI CpG methyltransferase (N.E.B) and then subjected to the bisulphite conversion process and subsequent DNA clean-up using the EpiMark Bisulpfite conversion kit (N.E.B). This DNA was PCR amplified using Epimark Hotstart Taq polymerase (N.E.B) and the primers APF BSP 5’- CGTGTTAGTTTAAGATGGTGAGG-3’ and APLR BSP 5’- CAAATCTCGAACCCGAAACACTA-3’, following the manufacturers recommended thermocycling program with an annealing temperature of 50°C and 35 cycles of amplification. The resultant PCR product was ligated into pCR4-TOPO-TA sequencing vector, cloned into TOP10 chemically competent *E*.*coli* (Invitrogen). The cloned vectors extracted and purified from overnight bacterial cultures using QIAprep Spin miniprep kit and finally sequenced to confirm methylation of CpG sites at -655/-654 and at -230/-229.

### Bioinformatics analysis of *AIRE/Aire* promoter region from different species

The sequence corresponding to exon 1 and the first 1kb upstream of exon 1 of AIRE in six different mammalian species (Human NM_000383.3; Chimpanzee XM_531580.3; Horse Q9Z0E3.1; Sheep XM_004003917.1; Pig XM_003358989.2; and Mouse XM_003358989.2) were aligned using the MAFFT alignment program using default parameters (Multiple Alignment using Fast Fourier Transform) [12] http://www.ebi.ac.uk/Tools/msa/mafft/ and S1 Fig.

Predicted transcription factor binding sites were identified using rVista 2.0 (all vertebrate matrices under “optimised for function” parameters) to analyse the human and chimpanzee sequences which had been aligned using zpicture [13] www.dcode.org.

## Results

### Screening for polymorphisms

**Fig 1:**
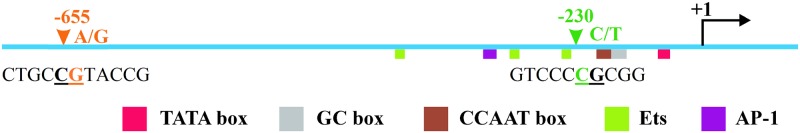
Important sequences and polymorphisms in *AIRE* promoter region. Scale Schematic representation of *AIRE* promoter haplotypes, indicating position of polymorphisms with immediate flanking sequence (potential CpG methylation site underlined) and reported positions of confirmed transcription factors found to bind to the *AIRE* promoter [11, 14].

We screened the first 591bp upstream of the *AIRE* transcription start site (TSS) in 32 human control DNA samples using transgenomic WAVE dHPLC (denaturing high performance liquid chromatography). We observed two different novel peaks which were identified by direct sequencing as the *AIRE*-655R (rs117557896) and *AIRE*-230Y (rs751032) polymorphisms, both of which have been reported in dbSNP. *AIRE*-230Y is found within the minimal AIRE promoter region [11], and is downstream of, but not within, a reported ETS-1 transcription factor binding site [14] (Fig 1). We subsequently used PCR RFLP (polymerase chain reaction restriction fragment length polymorphism) assay to screen our human control DNA samples to identify the genotype for *AIRE*-655R SNP, and complement the genotype data we had previously obtained for the *AIRE*-230Y SNP [10].

### *AIRE-230* and *AIRE-655* polymorphisms allele frequency

To determine the allelic distribution of *AIRE-*230 and *AIRE*-655 polymorphisms we genotyped 161 control Caucasian samples for the two polymorphisms (S1 Table). We found that *AIRE*-655A allele is extremely rare (0.01) whereas *AIRE*-230 was informative with the frequency of *AIRE*-230T allele of 0.10. The 1000 genomes project [15] does not have population frequency data for *AIRE*-655R, but our data for *AIRE*-230Y is in keeping with the 1000 genomes project data, with the C allele reported at a frequency of 0.885 and the T allele at 0.115 within the GBR sub-population. These frequencies are almost identical to the overall allele frequencies when accounting for all populations. However, there are differences in allele frequencies between the reported subpopulations, with the African population reported as C:0.995 T:0.006 at one end of the scale and the Asian population as C:0.718 T:0.282 on the other end.

In our samples, the common haplotype was *AIRE*-655G *AIRE*-230C with frequency of 0.89 whereas *AIRE*-655G *AIRE*-230T and *AIRE*-655A *AIRE*-230C have frequencies of 0.10 and 0.01 respectively. *AIRE*-655A *AIRE*-230T haplotype was not found in our cohort and therefore not included the reporter gene assay.

### Generating single integrated vector reporter gene assays

Reporter gene assays are commonly used to assess the effect of polymorphisms on the activity of genetic regulatory regions. Unfortunately, when performing these assays using transient transfection of the reporter vector, there are many variables that can influence the observed activity besides the genetic variation under investigation [16]. Therefore we decided to develop a reporter gene assay using the Flp-In system of site-specific recombination to generate recombinant cell lines containing a single copy of the reporter vector stably integrated into a defined position in the host cell line genome.

### Reporter gene assays

**Fig 2:**
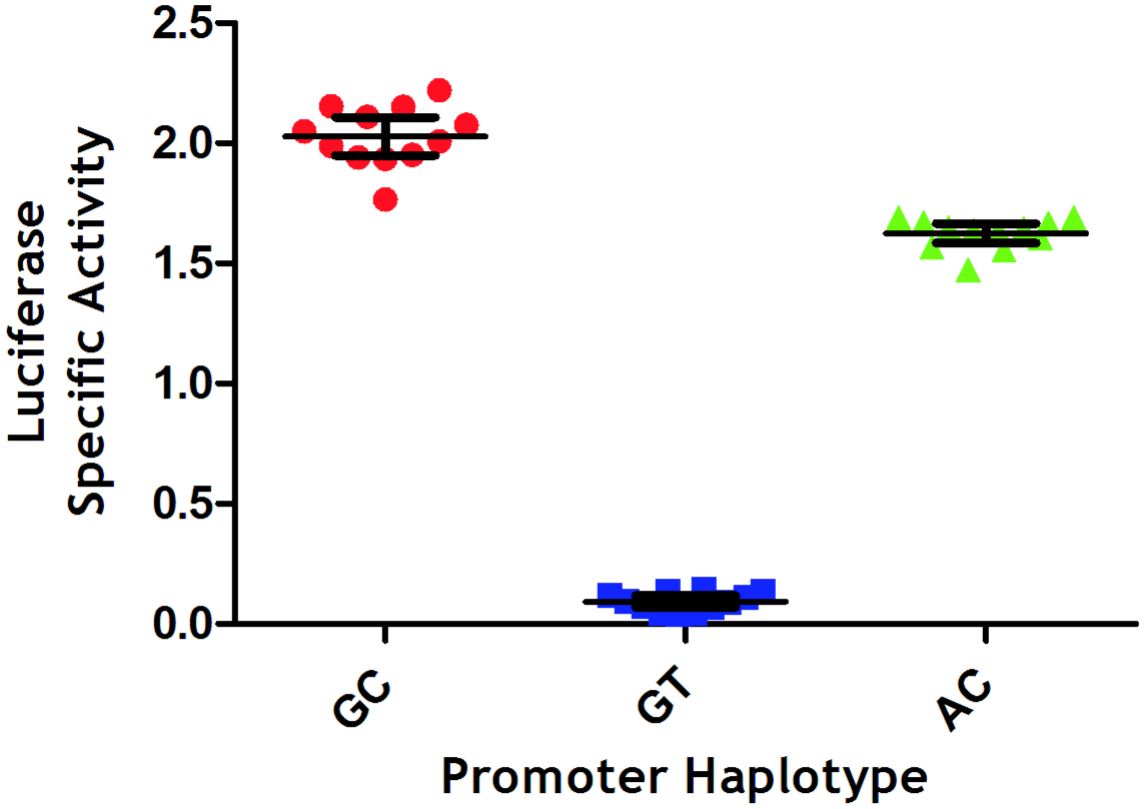
Luciferase specific activity for each *AIRE* promoter haplotype. Luciferase specific activity data (mean with 95% confidence intervals) obtained for 12 cell lysates of each different AIRE-promoter haplotype/firefly luciferase cell line generated using the integrated reporter assay system, and found to be significantly different between the different promoter haplotypes (P<0.001, Tukey-Kramer multiple comparison test). Lysates were assayed in triplicate with specific activity of each lysate calculated as normalised luminescence/protein concentration in μg/ml.

After introducing the different *AIRE* promoter/firefly luciferase plasmid construct into the host TEC 1A3 Flp-In cell lines, relative promoter activity was estimated by comparing the luciferase specific activity (normalised luminescence/protein concentration) for 12 lysates of each different reporter cell lines (Fig 2). One-way ANOVA was used to analyse mean luciferase specific activity and indicated that there was a significant difference in promoter activity (P<0.05), with a significant difference in activity between each promoter haplotype (P<0.001) also identified using the Tukey-Kramer multiple comparison test. The activity levels for the AC promoter haplotype was approximately 20% lower than for the GC promoter haplotype, with the activity for the GT haplotypes being over a factor of 10 lower than the GC haplotype. These results suggest that both the *AIRE*-655R and the *AIRE*-230Y polymorphisms influence *AIRE* promoter activity, with the *AIRE*-230Y polymorphism, found within the *AIRE* minimal promoter region, having a greater effect.

### Methylation and bisulphite sequencing of the *AIRE* promoter

As the demethylation of the *AIRE* promoter has been shown to influence AIRE expression in TEC 1A3 cells [11] we performed methylation assay using the M.SssI CpG methyltransferase. Both polymorphisms affect CpG sites with the least common allele of both polymorphisms (-655A and -230T) abrogating the CpG sequence. The alleles with an intact CpG sequence are also the most frequent allele for each SNP, but neither had been investigated in an earlier studies (-655G and -230C) [11, 17]. We therefore confirmed that the intact CpG site for each SNP could be methylated via bisulphite sequencing of the GC haplotype variant of our *AIRE* promoter/firefly luciferase reporter gene plasmid vector construct that had been treated with the CpG methyltransferase, M.SssI. Our results showed that both AIRE promoter sites can be methylated.

### *AIRE* promoter sequence in other species

**Fig 3:**
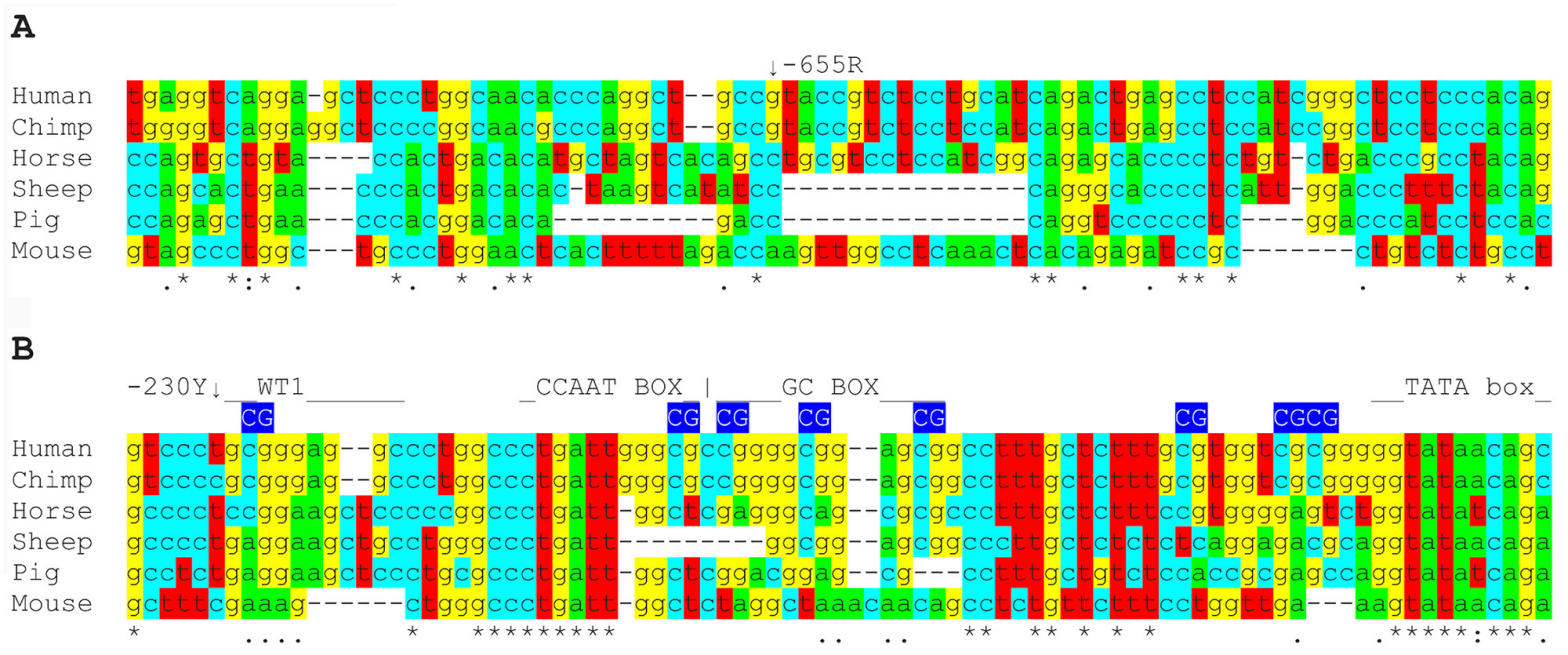
Regions of human AIRE promoter containing tested SNPs, and their alignment with equivalent regions from six other species. Sections of the MFATT alignment of AIRE promoter region in six mammalian species, indicating the positions of the *AIRE*-655 (A) and *AIRE*-230 polymorphisms, as well as showing the relative positions of nearby CpG sites on the human AIRE promoter that have been investigated [11, 14] as well as transcription factor binding sites identified for this sequence.

The 5’ region (encompassing the promoter) upstream of AIRE was compared across six different mammalian species by multiple sequence alignment using MFATT (Fig 3 and S1 Fig). This revealed that the region surrounding the AIRE-655R SNP had poor cross-species genetic conservation, whereas the region surrounding the AIRE-230Y SNP displayed much higher cross-species sequence conservation, with the nearby CAATT box and TATA box transcription factor binding sites clearly conserved across all analysed species. The sequence immediately downstream of AIRE-230Y was well conserved across the different species, suggesting there may be a transcription factor binding site (TFBS) at this position that has not previously been reported. Analysis of the AIRE promoter region using rVista 2.0 [13] indicated a predicted TFBS for WT1 (Wilms Tumour 1) at this position with the predicted binding site immediately downstream of -230Y.

## Discussion

In this study we developed a reporter gene assay system to investigate polymorphisms in the region immediately upstream of the *AIRE* gene, and determined the effect on transcriptional activity for the different alleles of two naturally occurring polymorphisms found within this region.

The use of a stably integrated reporter gene assay system has minimised any effect on our final results that might have been caused by experimental variables such as the amount of DNA; the amount of transfection control vector; the method of transfection; and the purity of transfected DNA, all of which have been reported to affect results from transient transfection reporter gene assays [16]. In addition, the presence of a unique integration site allows us to have an accurate system where we can create different cell line variants which only differ by the variation specifically incorporated into the reporter vector. Consequently, we can be confident that the significantly different levels of expression observed are due to the genetic variation under investigation and not experimental artefacts.

Whilst both the rarer -655A (AC promoter) and -230T (GT promoter) alleles caused a reduction in luciferase expression compared to the expression observed for the most frequent alleles (GC promoter) for these SNPS, it was the -230T allele that had the greatest effect, with luciferase specific activity reduced by approximately 95%. As the -230Y SNP and not the -655R SNP is found within the minimal *AIRE* promoter region, it is not entirely unsurprising that this polymorphism would alter expression the most, we would expect a partial reduction in AIRE expression comparable to those observed in mice as a result of the -230T allele, especially for individuals homozygous for -230T. In fact, phenotypic differences, in terms of incidence of autoimmune activity and changes in PTA expression, have been observed in murine studies where Aire expression has been partially reduced (50–63%) [7–9]. We would therefore consider that the *AIRE* -230Y SNP has the potential to affect AIRE regulated promiscuous gene expression, and consequently have an effect of negative selection and autoimmune susceptibility. It is also important to mention that the highest *AIRE* promoter activities are given by the commonest haplotype *AIRE*-655G *AIRE*-230C (Fig 2). However, whilst the lowest *AIRE* promoter activity was associated with *AIRE*-655G *AIRE*-230T and was found in 10% of the control cohort, none were homozygous for *AIRE*-655G *AIRE*-230T. Interestingly, we looked at this haplotype in 172 patients with alopecia areata, an autoimmune skin disorder associated with APS-1 and found 4 patients homozygous for this haplotype, suggesting that *AIRE*-655G *AIRE*-230T could be a susceptibility haplotype for alopecia areata outside the context of APS-1, but a larger cohort is need for confirmation [10].

The -230Y SNP is located in a gap in the promoter sequence that lies between two confirmed transcription factor binding sites (upstream of a NF-Y binding site and downstream of an Ets binding site) [11, 14]. This SNP lies next to a region of sequence that is well conserved over many different mammalian species and we identified a potential WT1 binding site, whose consensus binding sequence is immediately downstream of -230Y. In addition, both cytosine and thymine were found at this position amongst the other species used in the alignment, suggesting that this polymorphism may not have originated in humans.

In addition to the change in sequence brought about by the -230Y SNP, there is a CpG methylation site (Fig 1) that is abolished by -230T allele and so may affect the methylation status of the *AIRE* promoter, especially as this site is adjacent to a CpG site that was found to be differentially methylated in different cell lines. This is of particular note, as demethylation of the *AIRE* promoter was found to play a major role in up-regulating the expression of AIRE in TEC 1A3 cells [11], yet in our TEC 1A3 cell reporter gene model, lower expression was seen in the alleles of both SNPs that abolished a CpG site. This therefore suggests that the different -230Y alleles affect AIRE expression by influencing the binding of biochemical factors via changes to consensus DNA binding sequence rather than via methylation status of the promoter.

These findings are in keeping with our previously identified genetic association between polymorphisms in the *AIRE* gene and susceptibility to alopecia areata and vitiligo, another autoimmune disorder associated with APS-1 [10, 18, 19]. More recently, a polymorphism in *AIRE* has been found to be a determinant for predisposition to rheumatoid arthritis in the Japanese population [20]. It is therefore evident for the need to undertake extensive genetic analysis of *AIRE* polymorphisms in large cohorts of patients with autoimmune diseases, in particular those associated with APS-1.

## Supporting Information

S1 Fig Complete MAFFT alignment of genomic sequences containing the first 1kb upstream from Exon 1 and Exon 1 of AIRE/Aire gene from six different species. ClustalW output displayed, with nucleotide specific colour highlighting added to the output. Above the alignments we have listed the tested SNPs, the CpG sites and confirmed TFBS that have been identified on the human sequence [11, 14]. The predicted WT1 binding site has also been added above the alignments.
(DOC)

S1 Table Allelic discrimination of *AIRE*-230 and *AIRE*-655 polymorphisms. 161 control samples were tested with frequencies of haplotypes formed by the two polymorphisms calculated.
(DOCX)

## References

[1] Derbinski J, Kyewski B. How thymic antigen presenting cells sample the body's self-antigens. Current opinion in immunology. 2010;22(5):592–600. 10.1016/j.coi.2010.08.003 20832274

[2] Anderson MS, Venanzi ES, Klein L, Chen Z, Berzins SP, Turley SJ, et al Projection of an immunological self shadow within the thymus by the aire protein. Science (New York, NY. 2002;298(5597):1395–401. . 12376594 10.1126/science.1075958

[3] Finnish-German APECED Consortium. An autoimmune disease, APECED, caused by mutations in a novel gene featuring two PHD-type zinc-finger domains. Nature genetics. 1997;17(4):399–403. . 9398840 10.1038/ng1297-399

[4] Nagamine K, Peterson P, Scott HS, Kudoh J, Minoshima S, Heino M, et al Positional cloning of the APECED gene. Nature genetics. 1997;17(4):393–8. . 9398839 10.1038/ng1297-393

[5] Betterle C, Greggio NA, Volpato M. Clinical review 93: Autoimmune Polyglandular Syndrome Type 1 The Journal of clinical endocrinology and metabolism. 1998;83(4):1049–55. . 9543115 10.1210/jcem.83.4.4682

[6] Betterle C, Zanchetta R. Update on autoimmune polyendocrine syndromes (APS). Acta Biomed. 2003;74(1):9–33. . 12817789

[7] Liston A, Gray DH, Lesage S, Fletcher AL, Wilson J, Webster KE, et al Gene dosage--limiting role of Aire in thymic expression, clonal deletion, and organ-specific autoimmunity. The Journal of experimental medicine. 2004;200(8):1015–26. . 15492124 10.1084/jem.20040581 PMC2211852

[8] Kont V, Laan M, Kisand K, Merits A, Scott HS, Peterson P. Modulation of Aire regulates the expression of tissue-restricted antigens. Molecular immunology. 2008;45(1):25–33. . 17599412 10.1016/j.molimm.2007.05.014 PMC1994210

[9] Oliveira EH, Macedo C, Donate PB, Almeida RS, Pezzi N, Nguyen C, et al Expression profile of peripheral tissue antigen genes in medullary thymic epithelial cells (mTECs) is dependent on mRNA levels of autoimmune regulator (Aire). Immunobiology. 2012; 218:96–104. 10.1016/j.imbio.2012.02.005 22564670

[10] Wengraf DA, McDonagh AJ, Lovewell TR, Vasilopoulos Y, Macdonald-Hull SP, Cork MJ, et al Genetic analysis of autoimmune regulator haplotypes in alopecia areata. Tissue antigens. 2008;71(3):206–12. 10.1111/j.1399-0039.2007.00992.x 18194361

[11] Murumagi A, Vahamurto P, Peterson P. Characterization of regulatory elements and methylation pattern of the autoimmune regulator (AIRE) promoter. The Journal of biological chemistry. 2003;278(22):19784–90. . 12651856 10.1074/jbc.M210437200

[12] Katoh K, Toh H. Recent developments in the MAFFT multiple sequence alignment program. Brief Bioinform. 2008;9(4):286–98. 10.1093/bib/bbn013 18372315

[13] Loots GG, Ovcharenko I. Dcode.org anthology of comparative genomic tools. Nucleic acids research. 2005;33(Web Server issue):W56–64. . 15980535 10.1093/nar/gki355 PMC1160116

[14] Murumagi A, Silvennoinen O, Peterson P. Ets transcription factors regulate AIRE gene promoter. Biochemical and biophysical research communications. 2006;348(2):768–74. . 16890195 10.1016/j.bbrc.2006.07.135

[15] Abecasis GR, Auton A, Brooks LD, DePristo MA, Durbin RM, Handsaker RE, et al An integrated map of genetic variation from 1,092 human genomes. Nature. 2012;491(7422):56–65. 10.1038/nature11632 23128226 PMC3498066

[16] Karimi M, Goldie LC, Cruickshank MN, Moses EK, Abraham LJ. A critical assessment of the factors affecting reporter gene assays for promoter SNP function: a reassessment of -308 TNF polymorphism function using a novel integrated reporter system. Eur J Hum Genet. 2009;17(11):1454–62. 10.1038/ejhg.2009.80 19471307 PMC2986691

[17] Kont V, Murumagi A, Tykocinski LO, Kinkel SA, Webster KE, Kisand K, et al DNA methylation signatures of the AIRE promoter in thymic epithelial cells, thymomas and normal tissues. Molecular immunology. 2011;49(3):518–26. 10.1016/j.molimm.2011.09.022 22036612

[18] Tazi-Ahnini R, McDonagh AJ, Wengraf DA, Lovewell TR, Vasilopoulos Y, Messenger AG, et al The autoimmune regulator gene (AIRE) is strongly associated with vitiligo. Br J Dermatol. 2008;159(3):591–6. 10.1111/j.1365-2133.2008.08718.x 18616774

[19] Tazi-Ahnini R, Cork MJ, Gawkrodger DJ, Birch MP, Wengraf D, McDonagh AJ, et al Role of the autoimmune regulator (AIRE) gene in alopecia areata: strong association of a potentially functional AIRE polymorphism with alopecia universalis. Tissue antigens. 2002;60(6):489–95. . 12542742 10.1034/j.1399-0039.2002.600604.x

[20] Terao C, Yamada R, Ohmura K, Takahashi M, Kawaguchi T, Kochi Y, et al The human AIRE gene at chromosome 21q22 is a genetic determinant for the predisposition to rheumatoid arthritis in Japanese population. Human molecular genetics. 2011;20(13):2680–5. 10.1093/hmg/ddr161 21505073

