# Supplementary material for: The AIRE -230Y Polymorphism Affects AIRE Transcriptional Activity: Potential Influence on AIRE Function in the Thymus
Source: PLoS One. 2015 May 15;10(5):e0127476. doi: 10.1371/journal.pone.0127476 (PMC4433237; doi:10.1371/journal.pone.0127476)
Supplement: S1 Fig — ClustalW output displayed, with nucleotide specific colour highlighting added to the output. Above the alignments we have listed the tested SNPs, the CpG sites and confirmed TFBS that have been identified on the human sequence [11, 14]. The predicted WT1 binding site has also been added above the alignments. (DOC) [file pone.0127476.s001.doc]

Human accccccctccaggccatgcctgcggggccaccacagcctcagcatcattgcaggcccca

Chimp -ccccccctccaggccatgcctgcggggccaccacagcctcagcatcattgcaggcccca

Horse ------------------------------------------------------------

Sheep tttgctggtatgctgttcccatctcatggaccaggggcccttgctgcgatcctccaggga

Pig -------------------------------catggcccacactcaaaatgcctgtcctt

Mouse ---------ttattattttcttttatgtgtttaagtgccttgcttgcatgatgtatattt

Human ggcctctgcacctggtcttgttttactgggggcactgtccccactcacgtccacctggga

Chimp ggtctctgcacctggtcttgttttactgggggcattgtccccactcacgtccacctggga

Horse ----------------------------------------------------cccaggaa

Sheep gccttcctcacttcccc---------------cagaagcttctgtcccccaccccaggaa

Pig gaagtcagctgccttct---------------aaaagctttatttctaacacacctgggg

Mouse ggtcaccacattcatgcagtgccagtaggagccaaag----------agggcatcagatg

. *:*. .

Human ccctcggctcctgtccactgcagctcccctccc-------------ggacaccttcccag

Chimp ccctcggctcctgtccactgcagctcccctcca-------------ggacaccttcccag

Horse -----gggtcctg---------------------------------gggtgcctctccct

Sheep -----gacccctg---------------------------------gggtgcctctccct

Pig -----gactcccaccggaatgggttcaattttagttttaatcttatgggtgccccaccct

Mouse ctctggaaccggagttacaaagggtt--------------------gtgagccatcctgt

*. * . * . .** *

Human atgcccccggaagctcctgtccaggccacagcatccctcagcctctgtcactggtcctag

Chimp atgcccccggaagctcctgtccaggccacagcatccctcagcctctgtcactggtcctag

Horse gggctgtgagccccacaaacacaggagctccc-tggctcatacacaggcatgtctgccca

Sheep ga---ctgagagccagccagacaggagctcccagggctaatgctcagacatgcctgtcca

Pig at------gaaagaacctgggcagggactg---aatctgagctgcagctacgacca--cg

Mouse gggtgttgagaaaca--agcccaagtgctctgcaagaggaacaagtgctcttaccactga

. ... .: . **.* . . * :* . .

Human gaagaccctttgggagctctcactcagggccacactcaggacccccgttgtggggctggc

Chimp gaagaccctttgggagctctcactccgggccacactcaggacccccgttgtggggctggc

Horse tggggtccgctg-----ccttctc-gaagcttgact------------------------

Sheep cagggttagtca-----ccttcctagaagctttact------------------------

Pig cgggatccttta-----acccact--gagctgggctgggg--------------------

Mouse gccatctctctggcccctctctttttaagttcagtgtgtgtgtgtgtgtgtgtgtgtg--

. . . * ..* .

Human cgccttccttctaaagcacctggaggaaggaaggagggggtcaatgcgagcctcaatccc

Chimp cgccttccttctaaagcacctggaggaaggaaggagggggtcaatgcgagcctcaatccc

Horse --------ttctaacacacctggaggaagcagagtagg---ttgcactggggtcc----c

Sheep --------tcccaacacacccggaggaggtagaggatggatcgaccctggggact----c

Pig --------gtcgaacccgcacctcctcagtgaatggagctgctgcagttgggtccttaac

Mouse --------tgtgtgtgcgtgtgcgtgcattctcgcttgcatatgtacatgtgtatggaat

:. *. .. * . * :.

Human caggcg---agtgtgccccttttaaagatgagggaaccgaggctcagag-aaggaaagga

Chimp caggcg---agtgtgccccttttaaagatgagggaaccgaggctcagag-aaggaaagga

Horse ccagca---aacgtgctcattttaaaggtgaggcgactgcagctcggagaaaggaaacga

Sheep ccagtg---gaaagtgtccatttaaagatga---------------gagaaggagagaca

Pig ccagtgcgccatgggggaactcctggaacgtggggactgaagctcagag-aggaaagtga

Mouse agtgaatatagggtgttttcttcaattgttcacctaacttctttttta--aaaaaattca

. * . . . * :. . * *....* *

Human cttgcctggcgtcacacagctagcctaagatggtgaggtcagga-gctccctggcaacac

Chimp cttgcctggcgtcacacagccagcctaagatggtggggtcaggaggctccccggcaacgc

Horse ctttcccagcctcacacagcctccaggcagcgcccagtgctgta----ccactgacacat

Sheep cttgccccacatcatccagcttccaggtgatgcccagcactgaa---cccactgacacac

Pig cttgctggacatcacctggcttccaggcaaggcccagagctgaa---cccacggacaca-

Mouse gtttctctgcgtagggtttctgtc------tgtgtagccctggc---tgccctggaactc

** * .* *.. * * * .* *:* . *. * .**

↓-655R

Human ccaggct--gccgtaccgtctcctgcatcagactgagcctccatcgggctcctcccacag

Chimp ccaggct--gccgtaccgtctcctccatcagactgagcctccatccggctcctcccacag

Horse gctagtcacagcctgcgtcctccatcggcagagcacccctctgt-ctgacccgcctacag

Sheep -taagtcatatcc---------------cagggcacccctcatt-ggaccctttctacag

Pig ---------gacc---------------caggtcccccctc----ggacccatcctccac

Mouse actttttagaccaagttggcctcaaactcacagagatccgc-------ctgtctctgcct

. * ** . . ** * . * *.

Human ccccaggc--gggcccctgagtaggggctctcagcttgtgtggaggtcccccaggaacac

Chimp ccccaggc--gggcccctgagtaggggctctcagcttgtgtggaggtcccccaggaacac

Horse cctcaggcctgggactctgagcaggggctgt--gcccac-----tcccccccggggaccc

Sheep ccccagactaaggattctgagcagcagctgc--ggaggc-----agcagtgcccactccc

Pig ccccaggtttgaggctccgagcagcagctgt--ggaggcgcccacgccccaccaggatgc

Mouse ccctagtgctggcactaaaggcgtgagccac-------cacactcagctttccatgtaat

** ** .. . ..* . .** . * :

Human caccccgatccagcctccatggaggctcttgcc--ggccactgggaggggccggtgcacc

Chimp caccccgatccagcctccatggaggctcttgcc--ggccactgggaggggccagtgcacc

Horse tgcccaaacccagtcccatggtggcctcttgccctggtctctctgacgtgccggacaccc

Sheep tgccccggctcagctccctggaggcttcctgcgctggcccctggcagctgctgga-----

Pig taccccagtgcag-cccatggagactccttgtcctggcctctctgagctgctggcggacc

Mouse attttgaggcaggttctatccctgaaccttgcttgagcttatccattcagttagttg---

.. ..* .: . * ** .* .* : * .*

Human ctgggcagcccctgccagggccctgagacccgagcct---ccccgccgagggcacctgtc

Chimp ctgggctgcccctgccagggccctgagacccgagcct---ccccgccgagggcacctgtc

Horse ggacaacccct---tccggtgcccaagacattggcctgggtgaggctaagggcatctgtc

Sheep ------------------------------------gcagctgtgtgaaggacacccagc

Pig tgagcagaccc---gcaggtgcctgagatgctcgggtgggtga--------acacctgtc

Mouse --------------cctggtcagtgagctctacagcc--------------actcctgtc

.*: * . *

_

Human tcggctttgccccattcgagcagggccctcgccgagg-----caggacagggccacattc

Chimp tcggctttgccccattcgagcagggccctcgccgagg-----caggacagggccacattc

Horse ctggctttcccccatttgaacagggccctgtgggatg-----caggccaggaccacattt

Sheep actgctttcccccatttgaacagtgccctgtgggatg-----ctggccggaaccacgttt

Pig ctggc-tccctccgtttgaacagggccccaagggatg-----ctgtccagaaccacattt

Mouse tttgcttccccacacaggcaatttgtccacagagccatctttcccgccagtcccggtttt

** * * .*. : *...: * ** *. . * .*.* **. **

__Ets-C__

Human --ggaagtgagagttctctgagtcccgcacagagcgagtctctgtccccagcccccaagg

Chimp --ggaattgagagttctctgagtcccgcacagagcgagtctctgtccccagcccccaagg

Horse tgggaactgagaggggtcccagccacctcactgagggatcagaagcttcctttacaaagg

Sheep gggaaattgag---------------------------tctagacgcttcttgaccacgg

Pig tgggaattgag---------------------------tcagg-----------------

Mouse taacccataaacagatcctctgtagggagactggagagacaactttcccgcttcctctat

. .. *.*. :*:

___AP1_____ __Ets-B_

CG CG CG

Human cagctgccctggtgggtgagtcaggccaggcccggagacttcccgagagcgagggaggga

Chimp cagctgccctggtgggtgagtcaggccaggcccggagacttcccgagagcgagggaggga

Horse aaagaaaagccgacagcctgctggcaggtgccgaatgggggctggcgggtggatgagacc

Sheep aaagaaaggtcagcagcctgccggaggggttcaa--gggcgctggcaggtggctgggacc

Pig -----------------------------------------------------tcgggcg

Mouse cccgaggccaggcagctgcactggatctcacaaagaaagagtatcagagcgt--------

_Ets-A_ -230Y↓__WT1______

CG CG

Human cagcagcgcctccatcacagggaagt----------gtccctgcgggag--gccctggcc

Chimp cagcagcgcctccatcacagggaagt----------gtccccgcgggag--gccctggcc

Horse ttctctttactcagttacagggaagtttcccttcctgcccctccggaagctcccccggcc

Sheep tt-ggggaactcagtcacggggaagt-----ttctggcccctgaggaagctgcctgggcc

Pig cggtcctctctcggttacagggaagtttcg-ttcctgcctctgaggaagctccctgcgcc

Mouse ---cctgaacccaaaggcagggcagtctctagcccagctttcgaaag------ctgggcc

* * .: .*.***.*** * .... * ***

_CCAAT BOX_|____GC BOX____ _TATA box

CG CG CG CG CG CGCG

Human ctgattgggcgccggggcgg--agcggcctttgctctttgcgtggtcgcgggggtataac

Chimp ctgattgggcgccggggcgg--agcggcctttgctctttgcgtggtcgcgggggtataac

Horse ctgatt-ggctcgagggcag--cgcgccctttgctctttccgtggggagtctggtatatc

Sheep ctgatt---------ggcgg--agcggcccttgctctctctcaggagacgcaggtataac

Pig ctgatt-ggctcggacggag--cg---cctttgctgtctccaccgcgagccaggtatatc

Mouse ctgatt-ggctctaggctaaacaacagcctctgttctttcctggttga---aagtataac

****** .. .. ** ** * * * . .*****:*

___

CG CGCGCG

Human agcggcgcgcgtg-----------------------------------------------

Chimp agcggcgcgcgtg-----------------------------------------------

Horse agagcccgggcct-----------------------------------ggctgctcagcc

Sheep agactccggccccgaggaggagccggagaagct----------ctcagggctccctctcc

Pig agagacccacccgcgcgctcggcccgggaggccgctcagcccgctctggtcccctctcca

Mouse agagtcgc----------------------------------------------------

**. *

CG CG CG C

Human ------------gctcgc-----------------agaccggggAGACGGGCGGGC----

Chimp ------------gctcgc-----------------agaccggggAGACGGGCGGGC----

Horse ccgcgtaggt--gcccgcccaggtgtgcccggcg-gggcaggaggagcgcgagggtcagg

Sheep ccgagcaggtgagcccgcccaggtgttcccggcgcaggcggagtgagtgcaagcaccagg

Pig ccagccaggtgtggccgcccagg---gccccgcg-gcgcagggcgagcgcgag-gccagg

Mouse -----tgagcaagacaaacgtcgcctttccgccg-ctggggagtaaatataaacgcttac

* ... . *.. ... . ... .

G CG CGCG CG CG CG CG

Human --GCACAGCCGGCGCGGAGGCCCCACAGCCCCGCCGGGACCCGAGGCCAAGCGAGGGGCT

Chimp --GCACAGCCGGCGCGGAGGCCCCACAGCCCCGCCAGGACCCGAGGCCAAGCGAGGGGCT

Horse aggcagacgtggcccggaca-cccgcaaatccagcggcacccagaggccgcgggacccca

Sheep aggcagacacggcccggacaccccacaaggccagcggcgccctgaggccccgagacccca

Pig aggcaggcacggcccagacatcccacaagtccagcgacaccccgaggccgcgggacccca

Mouse aggtgcctct-----------ccggatagcaccacgacacccaaggaagggagaaggGAA

* . ** ..:. .* *.. .*** ..* . ... .:

CG CGCG CG CG CGCG

Human GCCAGTGTCCCGGGACCCACCGCGTCCG--CCCCAG---CCCCGGGTCCCCGCGCCCACC

Chimp GCCAGTGTCCCGGGACCCACCGCGTCCG--CCCCAG---CCCCGGGTCCCCGCGCCCACC

Horse ggcggggagagggggcccccagcgccgcg-ccccagaacccgcgatggccagcgaggccc

Sheep ggcggggagagggggcccccagcgctgcgtccccggaccccgcgATGGCGGGCGAGGCCC

Pig ggcggggagagggagcccccagccccgcgaccccgggccccacgatggcgggcgaggccC

Mouse CGCAAGCGCGCGTGGGC--CAGCAGGGGGCGCCGAGGCGCAGCCCCTGTGAGGAAGAT--

*.. * .. * *.** ** .* *. * * ..

CG CG CGCG CG CG CG CG CG CGCG

Human CCATGGCGACGGACGCGGCGCTACGCCGGCTTCTGAGGCTGCACCGCACGGAGATCGCGG

Chimp CCATGGCGGCGGACGCGGCGCTACGCCGGCTTCTGAGGCTGCACCGCACGGAGATCGCGA

Horse ggGCCGGCGGGGATGCGTCGCTGCGCCGCCTCCTGAGGCTGCACCGCACCGAGATCGCCG

Sheep GGGCCGGTGGGGACGCGGCGCTGCGCCGCCTGCTGAGGCTGCATCGCACGGAGATCGCGG

Pig GTGCGGGTGGGGACGCCGCGCTGCGCCGCCTCCTGAGGCTGCACCGCACGGAGATCGCGG

Mouse -GGCAGGTGGGGATGGAATGCTACGCCGTCTGCTGAGGCTGCACCGCACCGAGATCGCGG

. * . *** * ***.***** ** *********** ***** ******** .

CG

Human TGGCCGTGGACAGCGCCTTCCCACTGCTGCACGCGCTGGCTGACCACGACGTGGTCCCCG

Chimp TGGCCGTGGACAGCGCCTTCCCACTGCTGCACGCGCTGGCCGACCACGACGTGGTCCCCG

Horse TGGCCGTGGACAGCGCCTTCCCGCTGCTGCACGCGCTGGCCGACCACGACGTGGTCCCCG

Sheep TGGCCGTGGACAGCGCCTTCCCGCTGCTGCACGCGCTGGCCGACCACGACGTGGTCCCCG

Pig TGGCGGTGGACAGCGCCTTCCCGCTGCTGCACGCGCTGGCCGACCACGAAGTGGTCCCCG

Mouse TGGCCATAGACAGTGCCTTTCCGCTGCTGCATGCTCTAGCCGACCACGACGTGGTCCCTG

**** .*.***** ***** **.******** ** **.** ********.******** *

Human AGGACAAGTTTCAG

Chimp AGGACAAGTTCCAG

Horse AGGACAAGTTCCAG

Sheep AGGACAAGTTCCAG

Pig AGGACAAGTTCCAG

Mouse AGGACAAGTTCCAG

********** ***

**S1 Fig. Complete MAFFT alignment of genomic sequences containing the first 1kb upstream from Exon 1 and Exon 1 of AIRE/Aire gene from six different species.** ClustalW output displayed, with nucleotide specific colour highlighting added to the output. Above the alignments we have listed the tested SNPs, the CpG sites and confirmed TFBS that have been identified on the human sequence [11, 14]. The predicted WT1 binding site has also been added above the alignments.
