# Supplementary material for: The AIRE -230Y Polymorphism Affects AIRE Transcriptional Activity: Potential Influence on AIRE Function in the Thymus
Source: PLoS One. 2015 May 15;10(5):e0127476. doi: 10.1371/journal.pone.0127476 (PMC4433237; doi:10.1371/journal.pone.0127476)
Supplement: S1 Table — 161 control samples were tested with frequencies of haplotypes formed by the two polymorphisms calculated. (DOCX) [file pone.0127476.s002.docx]

| ***AIRE* promoter polymorphisms** | **Frequency** |
| --- | --- |
| *AIRE*-655G | 0.99 |
| *AIRE*-655A | 0.01 |
| *AIRE*-230C | 0.90 |
| *AIRE*-230T | 0.10 |
| *AIRE*-655G *AIRE*-230C | 0.89 |
| *AIRE*-655G *AIRE*-230T | 0.10 |
| *AIRE*-655A *AIRE*-230C | 0.01 |
| *AIRE*-655A *AIRE*-230T | Not found |

**S1 Table. Allelic discrimination of *AIRE*-230 and *AIRE*-655 polymorphisms**

161 control samples were tested with frequencies of haplotypes formed by the two polymorphisms calculated.
